# Data-driven discovery of seasonally linked diseases from an Electronic Health Records system

**DOI:** 10.1186/1471-2105-15-S6-S3

**Published:** 2014-05-16

**Authors:** Rachel D Melamed, Hossein Khiabanian, Raul Rabadan

**Affiliations:** Department of Systems Biology and Department of Biomedical Informatics; Columbia University College of Physicians and Surgeons, New York, NY, United States

## Abstract

**Background:**

Patterns of disease incidence can identify new risk factors for the disease or provide insight into the etiology. For example, allergies and infectious diseases have been shown to follow periodic temporal patterns due to seasonal changes in environmental or infectious agents. Previous work searching for seasonal or other temporal patterns in disease diagnosis rates has been limited both in the scope of the diseases examined and in the ability to distinguish unexpected seasonal patterns. Electronic Health Records (EHR) compile extensive longitudinal clinical information, constituting a unique source for discovery of trends in occurrence of disease. However, the data suffer from inherent biases that preclude a identification of temporal trends.

**Methods:**

Motivated by observation of the biases in this data source, we developed a method (Lomb-Scargle periodograms in detrended data, LSP-detrend) to find periodic patterns by adjusting the temporal information for broad trends in incidence, as well as seasonal changes in total hospitalizations. LSP-detrend can sensitively uncover periodic temporal patterns in the corrected data and identify the significance of the trend. We apply LSP-detrend to a compilation of records from 1.5 million patients encoded by ICD-9-CM (International Classification of Diseases, Ninth Revision, Clinical Modification), including 2,805 disorders with more than 500 occurrences across a 12 year period, recorded from 1.5 million patients.

**Results and conclusions:**

Although EHR data, and ICD-9 coded records in particular, were not created with the intention of aggregated use for research, these data can in fact be mined for periodic patterns in incidence of disease, if confounders are properly removed. Of all diagnoses, around 10% are identified as seasonal by LSP-detrend, including many known phenomena. We robustly reproduce previous findings, even for relatively rare diseases. For instance, Kawasaki disease, a rare childhood disease that has been associated with weather patterns, is detected as strongly linked with winter months. Among the novel results, we find a bi-annual increase in exacerbations of myasthenia gravis, a potentially life threatening complication of an autoimmune disease. We dissect the causes of this seasonal incidence and propose that factors predisposing patients to this event vary through the year.

## Background

Temporal patterns in human disease often reflect changing environmental factors, as is evident in levels of allergic disease in spring and fall, vector-borne and enteric diseases in summer, and respiratory infectious diseases in winter. Thus, discovering temporal associations can potentially inform us of unconsidered causes of a wide variety of human diseases. As Electronic Health Records (EHR) increasingly compile clinical information from large numbers of patients in a computationally accessible form, they represent a unique opportunity to seek these patterns. When this data is explored with appropriate methods, unbiased discovery of trends in incidence could illuminate a diverse array of health conditions.

The distribution of disease events over time has been used to distinguish characteristics unique to that disease. For example, our group has compared the typical annual winter increase in influenza diagnoses against the 2009 influenza pandemic, identifying an unusual timing of peak influenza hospital admissions in the pandemic [1]. Seasonality is known to be a strong influence in the occurrence of viral infections, which are more likely in the winter; bacteria, on the other hand have been reported to opportunistically infect in warmer weather [2]. Thus, scanning EHR data for signals in the incidence of disease over time could be informative as to pathogenic involvement as well as other influencing factors in the lead-up to hospital visit.

Recently, temporal clustering of cases of disease has been examined for a variety of purposes. In an effort to characterize Kawasaki disease, a childhood vasculitis with potential for serious cardiac complication, Burns, as well as Rodó, have examined the spatiotemporal distribution of cases [3,4]. These breakthrough findings have suggested that the thus far unidentified causative pathogen may be a windborne microbe. Other groups have sought to confirm anecdotal findings of seasonal or climate related changes in disease rates, including heart failure [5], depression and anxiety [6], varicose vein ulcers [7], urinary tract infection [8,9], and even cancer [10]. While some of these works searched for seasonality using purpose-driven surveys, Upshur [11] used coded administrative data derived from a large EHR system to investigate whether seasonal peaks in incidence were a common feature in a limited set of the most frequent diagnoses. Some of these findings emphasize the behavioral causes of seasonal changes in hospital visits, underlining the importance of attributing the likely biological versus sociological causes of the patterns.

The EHR is a rich source of data, including free text entries as well as coded terms, such as the diagnosis coding system ICD-9-CM (International Classification of Diseases, Ninth Revision, Clinical Modification). ICD-9 codes are entered into the record for administrative purposes and may not capture all cases, but they have been shown to have a strong predictive value in a wide range of diseases, including skin infection [12], urinary tract infection [13], acute myocardial infarction [14], and chronic obstructive pulmonary disease [15]. As ICD-9 is an international standard, it also suggests that a uniform methodology could be applied across EHR data from multiple systems. We and others have successfully mined diagnosis codes for factors underpinning disease. Finding significant disease comorbidities using ICD-9 codes has had implications for the underlying genetic factors of some diseases [16] and has been used to suggest unforeseen causes or consequences of disease [17]. Changes in ICD-9 code incidence over time have been utilized in isolating the driving cause of increased burden to emergency departments over time [18], and for examining trends in high-risk surgical procedures[19], among other studies. By seeking periodically repeating patterns in the distribution of diagnosis over time, we have chosen a research question that is resistant to many of the biases inherent in ICD-9 codings.

Despite the potentially strong statistical power of these large compilations of patient records, multiple confounding factors preclude a reliable identification of periodic trends. In the New York-Presbyterian EHR system, we observe that incidence of diagnoses often increases over the years, and the overall number of hospitalizations displays seasonal changes. In this manuscript, we propose a method (Lomb-Scargle periodograms in detrended data, LSP-detrend) to adjust for these biases and robustly identify periodic temporal patterns using Lomb-Scargle periodograms, a method originally developed in astronomy. We have undertaken the first comprehensive survey of seasonality in ICD-9 code incidence, accounting for these biases in this data. We have thus quantified the strength of seasonal trend in the 2,805 most common diagnosis codes used over a 12 year period in the New York-Presbyterian EHR system. Researching the resulting seasonal effects in the literature, we determined which of these phenomena confirm reported or well known trends, and which are novel. Among the interesting results, we find a bi-annual increase in acute exacerbations of myasthenia gravis (ICD-9 code 358.01), in late winter and late summer. As acute exacerbation is a serious complication of myasthenia gravis, this discovery could be significant for the understanding of this disease. Thus, we searched the EHR for clues as to the cause of this seasonal pattern, using ADAMS [17] to identify diagnoses that are comorbid with the exacerbations in myasthenia gravis. This work points to the potential of the EHR as a source for unbiased pattern discovery, with implications for understanding human disease.

## Results

### LSP-detrend: finding periodic signal

The New York-Presbyterian hospital's EHR system contains health information for 1.5 million patients, collected over the last three decades and including both free text and coded entries for diagnosis (ICD-9), procedures, prescription orders, and lab results. We selected the time period 1997 to 2009 because the number of entries before 1997 was sharply decreased in comparison. After identifying the 2,805 diagnoses with more than 500 cases over the time period, we extract diagnosis date and patient identifier for all patients with the diagnosis, and create a count of the number of unique patients diagnosed every month.

**Figure 1:**
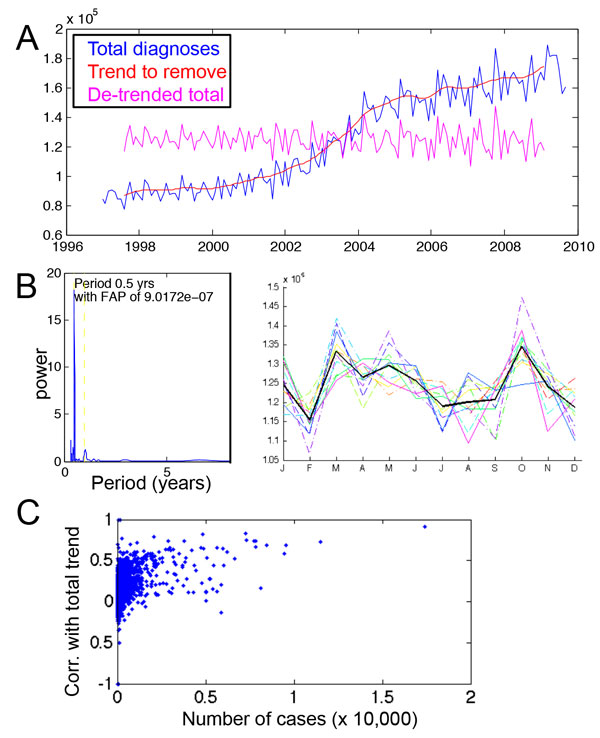
**Identifying confounding factors in temporal diagnosis**. A. Aggregated number of diagnoses from 1997 to 2009 (blue) show a strong increasing trend over time, modeled by the red line. When this trend is subtracted out, the remaining signal (magenta) shows no overall trend but a seasonal trend. B. De-trended total diagnoses display 6 month periodicity, as shown by the periodogram. When the years are plotted on top of each other (each colored line represents a year, with the bold black line the average), the spring and fall show consistent peaks in diagnosis each year. C. For each diagnosis, the number of cases is compared with the seasonal pattern in incidence. The most frequently occurring diagnoses show the most correlation with the overall spring-fall peak incidence; the overall trend causes false detection of periodic signal. Correlation between number of cases (x-axis) and correspondence with the overall spring-fall trend (y-axis) is 0.41.

Two trends immediately emerge on considering all codes in aggregate. First, the number of patients visiting the hospital for any reason clearly increases over time (Figure 1A). We remove this trend for each code by subtracting out a smoothed version of the incidence information, a procedure we call de-trending. Upon removing this trend, a seasonal increase in the number of hospital visits in the spring and in the fall appears (Figure 1B). This is reflected in the monthly frequencies of the most common diagnoses: the more common a diagnosis is, the more its monthly incidence reflects this overall trend (Figure 1C). Some of the most common are chronic diseases, such as Unspecified Essential Hypertension (401.9), Obesity unspecified (278.00), and Osteoporosis unspecified (733.00). We believe that the high prevalence of chronic diseases in the set of disorders with fall and spring peaks indicates that these diagnoses are not actually the drivers of hospital visits. More likely, hospital visits increase seasonally for a number of reasons and these common diseases simply represent a fixed proportion of the overall population. Thus, we remove the total population trend as well, in a procedure that we call de-totaling.

The final step of LSP-Detrend assesses the presence of periodic signal in the adjusted data. Lomb-Scargle periodograms have a number of advantages in detecting periodic events. In this method, the time series of monthly rate of diagnosis is the input, and for a range of possible periods, the power of that period, and an associated statistical significance, is calculated. The period of greatest power is calculated for the data, the de-trended data, and the de-trended and de-totaled data.

### Major types of periodic signal and known seasonal disease

**Figure 2:**
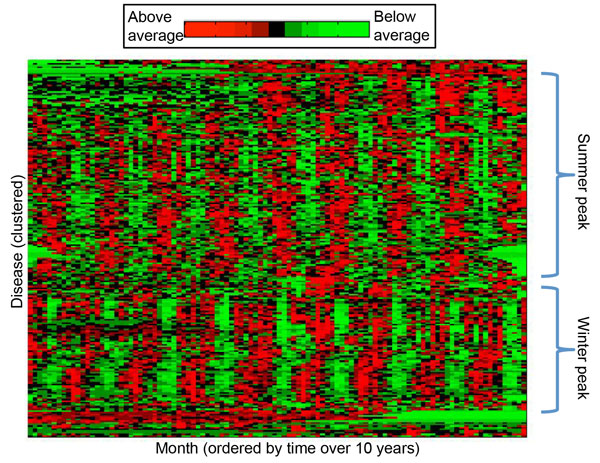
**Pre-processed and row-normalized monthly incidence for 227 codes with periodic signal**. Each row is a disease, and each column a month over 10 years. Thus, boxes in a row represent incidence of that disease for each month, with red signifying elevated incidence and green decreased incidence. Two main clusters stand out: diseases that occur in the summer (top), and those that occur in winter.

Of the 2,805 codes examined, we identify 284 with periodic signal that could represent seasonal peaks in incidence (Supp. Table 1). We perform hierarchical clustering of the monthly occurrence of each disease to look for groups of conditions with similar period and phase of incidence (Figure 2). The clustering shows that two main groups comprise most of the codes, containing events that occur in winter (such as viral infections and respiratory infections), and those that occur in summer (mostly fractures and wounds). Based on the groupings of codes, seasonal influences appear to arise from a number of sources.

**Table 1:** Conditions associated with myasthenia gravis with acute exacerbation As described in the text, this table details conditions associated with the exacerbation as compared with two controls, influenza and hip joint pain, within two months of exacerbation.

ICD-9 description	Have disease (case set, 204 patients)	Incidence (case set)	Have disease (control set, 2582 patients)	Incidence (control set)	Odds ratio	p-value
Carpal tunnel syndrome	9	0.044118	2	7.75E-04	56.96	2.47E-09
Urinary tract infection site not specified	26	0.127451	76	0.029435	4.33	5.95E-09
Pneumonitis due to inhalation of food or vomitus	15	0.073529	21	0.008133	9.041	7.94E-09
Anxiety state unspecified	14	0.068627	22	0.008521	8.054	7.39E-08
Unspecified essential hypertension	53	0.259804	305	0.118125	2.199	9.73E-08
Esophageal reflux	19	0.093137	56	0.021689	4.294	8.60E-07
Unspecified pleural effusion	12	0.058824	21	0.008133	7.232	1.55E-06
Friedlander\'s bacillus infection in conditions classified elsewhere and of unspecified site	6	0.029412	2	7.75E-04	37.97	3.55E-06
Thyrotoxicosis without goiter or other cause and without thyrotoxic crisis or storm	6	0.029412	2	7.75E-04	37.97	3.55E-06
Atrial fibrillation	15	0.073529	41	0.015879	4.631	6.17E-06
Other specified disorders of pancreatic internal secretion	4	0.019608	0	0	-1	2.80E-05
Other specified idiopathic peripheral neuropathy	4	0.019608	0	0	-1	2.80E-05
Pure hypercholesterolemia	20	0.098039	83	0.032146	3.05	3.49E-05
Hemorrhage complicating a procedure	5	0.02451	2	7.75E-04	31.64	3.73E-05
Long-term (current) use of steroids	5	0.02451	3	0.001162	21.09	9.37E-05
Personal history of noncompliance with medical treatment presenting hazards to health	7	0.034314	13	0.005035	6.815	3.47E-04
Hematoma complicating a procedure	4	0.019608	2	7.75E-04	25.31	3.73E-04
Adrenal cortical steroids causing adverse effects in therapeutic use	5	0.02451	5	0.001936	12.66	3.73E-04
Nontoxic uninodular goiter	3	0.014706	0	0	-1	3.87E-04
Chronic lymphocytic thyroiditis	3	0.014706	0	0	-1	3.87E-04
Personal history of malignant neoplasm of bladder	3	0.014706	0	0	-1	3.87E-04
Personal history of malignant neoplasm of other endocrine glands and related structures	3	0.014706	0	0	-1	3.87E-04
Embolism and thrombosis of other specified veins	4	0.019608	3	0.001162	16.88	8.20E-04
Depressive disorder not elsewhere classified	14	0.068627	62	0.024012	2.858	9.55E-04
Toxic diffuse goiter without thyrotoxic crisis or storm	3	0.014706	1	3.87E-04	37.97	0.001465
Unspecified idiopathic peripheral neuropathy	3	0.014706	1	3.87E-04	37.97	0.001465
Unspecified disorder of optic nerve and visual pathways	3	0.014706	1	3.87E-04	37.97	0.001465
Personal history of tobacco use	8	0.039216	24	0.009295	4.219	0.001632
Diabetes mellitus without complication type i not stated as uncontrolled	8	0.039216	25	0.009682	4.05	0.002022
Hypertrophy (benign) of prostate without urinary obstruction	5	0.02451	9	0.003486	7.032	0.002323
Bipolar disorder, unspecified	4	0.019608	5	0.001936	10.13	0.002624
Unspecified disorder of thyroid	3	0.014706	2	7.75E-04	18.99	0.003465
Retention of urine unspecified	3	0.014706	2	7.75E-04	18.99	0.003465
Other specified retention of urine	3	0.014706	2	7.75E-04	18.99	0.003465
Migraine unspecified without mention of intractable migraine without mention of status migrainosus	4	0.019608	6	0.002324	8.438	0.004125
Other pulmonary embolism and infarction	4	0.019608	6	0.002324	8.438	0.004125
Unspecified sleep apnea	4	0.019608	6	0.002324	8.438	0.004125
Anemia unspecified	11	0.053922	53	0.020527	2.627	0.005828
Methicillin susceptible staphylococcus aureus	3	0.014706	3	0.001162	12.66	0.006558
Obstructive sleep apnea (adult)(pediatric)	3	0.014706	3	0.001162	12.66	0.006558
Tracheostomy status	4	0.019608	10	0.003873	5.063	0.015584

Only the 209 patients with other conditions in the preceding two months are considered, and the number of patients with the associated condition is in the column "Have disease (case set)", with the incidence rate in the following column. The control set shown is influenza, with analagous columns. Finally the odds ratio and statistics are shown. We filter out any conditions that are directly associated with myasthenia gravis (such as shortness of breath), and also any conditions occurring in fewer than three patients with the disease.

Clearly, behavior changes associated with different times in the year explain many of the patterns, such as the predominance of accidents in the summer. Rashes and skin infections like impetigo can be linked to more skin exposure in the summer, and the same factor most likely explains the increased diagnosis of the chest bone malformity pectus excavatum (code 754.81). Seasonal behavior change also drives the pattern in diagnoses pertaining to child psychiatric disorders, including attention deficit disorder and adjustment disorders, which dip sharply during the summer school break. Another well known annual pattern, the increase in births in the summer, has also been suggested to be most attributable to behavior, though other factors may play a role [20].

Although behavior explains many of the changes in diseases rates throughout a year, environmental risk factors clearly vary as well, including allergens, ultraviolet light, and the virulence of pathogens. Thus, it is expected that seasonal allergies, influenza, pneumonia, scarlet fever, and complications from these disease have clear peaks in incidence. It is reassuring that all of these effects were captured in our data and by LSP-detrend. We have focused on the findings that appear most novel, interesting, and interpretable. All results are available in Additional File 1.

### Confirmation of recent reports of seasonal effects

Often driven by anecdotal reports of increased rates of a disease in certain times of the year, a number of groups have sought to scientifically assess the presence of these patterns. They have used a variety of methods suited to their purposes, such as questionnaires, mining surveillance databases, examining lab results, or chart review for a pre-selected set of patients. Our work, on the other hand, does not require one particular hypothesis, but instead it is suitable to ranking the seasonality for all diagnoses in the hospital, using already existing data. Instead we take advantage of an already rich data source, and we show that LSP-detrend, and the ICD-9 data, is able to reproduce these findings. In researching the diseases with strongest signal, we came across a number of previous reports of periodic disease incidence, and we compare our findings to them.

**Figure 3:**
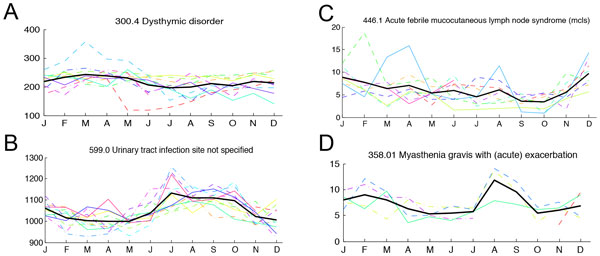
**Selected diseases with periodic signal**. For four of the diseases discussed, the monthly incidence for all years is plotted together in order to view consistencies in the seasonal trend across different years. Each colored line again represents a year, with the bold black line the average across the years.

Recent reports have found seasonal changes in occurrence of anxiety and depression [6] and seasonality of key neurotransmitters involved in mood has been documented [21,22]. Our analysis showed a strong winter and early spring increase in obsessive-compulsive disorder (300.3), dysthymic disorder (300.4), shown in Figure 3A, and other depressive disorders (311). It is difficult to attribute trends in these complex disorders to behavioral versus environmental influences, but interestingly, we find that many other psychiatric disorders, such as dependent personality disorder and social phobia, had no seasonal pattern.

Other periodically increasing diseases can be more clearly attributed to seasonally increased environmental risk. A number of recent reports have asserted that bacterial infections are more frequent in warmer weather generally [2], that season has a significant effect on bacteria exposure [23], and that bacterial bloodstream infections peak in summer [24]. Our data strongly support the hypothesis that bacteria are more virulent in the summer. We detect strong summer peaks in urinary tract infection (code 599.0), shown in Figure 3B, and its complications of pyelonephritis (590.10 and 590.80), and hematuria (599.7). This corroborates the results of [8,9]. We also found increased rates of cellulitis and abscess in the summer months. Other groups have found increased incidence of soft tissue infections in the summer [25]. Finally, there is a strong late summer peak incidence of vascular device inflammation and infection (code 996.62), which may be due to the same influences.

Finally, as we were initially inspired to undertake this work in order to search for new pathogenic effects contributing to disease incidence, we found the detection of an annual winter peak in Acute febrile mucocutaneous lymph node syndrome (mcls) (code 446.1), also known as Kawasaki disease, to be particularly interesting. The winter peak in incidence (Figure 3C) is strong and consistent with previous US findings [4]. This is a somewhat rare disease in our study population, yet LSP-detrend is able to confidently identify the pattern.

Thus, our results contribute to the state of knowledge of a range of human diseases, and many of our findings are buttressed by previous reports investigating specific hypotheses about disease incidence.

### Novel findings: acute exacerbations of myasthenia gravis

One of our findings stands out as a well-defined diagnosis of a highly acute condition that has a previously entirely unreported seasonal incidence: myasthenia gravis with acute exacerbation, code 358.01. Although this is a rare condition, the seasonal trend is strong, and is visible in Figure 3D, with peak incidences in late winter and in late summer.

Myasthenia gravis is an autoimmune disease largely characterized by presence of antibodies targeting elements of the nerve to muscle junction, resulting in blocked neural signals to the muscle and subsequent muscle weakness [26]. Subtypes include patients with antibodies targeting the acetylcholine receptor, and against the muscle specific kinase receptor, with variable phenotype, including treatment response, depending on the category of autoimmune antibody. Patients, usually older middle-aged people or young women, often primarily present with eyelid weakness (ptosis) or difficulty swallowing (dysphagia) or other signs of weakness. Suggested underlying causes include abnormalities of the thymus, such as thymoma, certain drugs, as well as a genetic component. Although immunosuppressive drugs or thymectomy can mitigate the weakness and allow a high quality of life for most subtypes of patients, the condition is incurable and is characterized by occasional acute episodes. Any infection can cause increased immune activity which can worsen an autoimmune condition, and the role of viral infections in particular have been investigated [27]. Other risk factors include stress, many drugs, and temperature, which directly reduces the efficiency of the neuromuscular junction. In acute exacerbation of myasthenia gravis, a patient experiences greater weakness, sometimes to the point where normal respiratory activity is threatened, resulting in respiratory failure. Thus, patients with this diagnosis are have a higher than average likelihood of seeking prompt treatment. The chronic condition, with a different administrative code, shows no seasonal pattern.

As this seasonal pattern has no obvious cause, we use the EHR data to search for common factors among patients with exacerbation. Concurrent diagnoses, prescriptions, or procedures could provide insight into cause of the seasonal pattern. Described previously [17], ADAMS, Application for Discovering Disease Associations using Multiple Sources, has been previously used with the same data set to find co-morbid diagnoses for rare diseases. We use the method to search for co-morbid events specific to the months preceding the exacerbation, and the results are shown in Table 1. When conditions that can be assumed to be a direct result of the myasthenia gravis are disregarded, the most associated diseases are urinary tract infection (code 599.0), carpal tunnel syndrome (code 354.0), unspecified essential hypertension (code 401.9) and esophageal reflux (code 530.81). We find it particularly interesting that both UTI and carpal tunnel are seasonally linked, although the explanation for the pattern in carpal tunnel is unclear. It is possible that treatment for a seasonally linked disease exacerbates the myasthenia gravis, as this condition is worsened by many commonly used drugs.

## Conclusions

Applying periodograms to administrative code data from EHR, we are able to identify a number of periodic patterns in an unbiased fashion. Importantly, we demonstrate the importance of examining the data for confounding trends. When the proper corrections are made, we confirm that LSP-detrend is sensitive to expected seasonal variation, and we also provide support for recent findings of seasonal distributions of disease. Most significantly among these, we find a pervasive pattern of increased incidence of bacterial infections in the warmer season, including urinary tract infection, cellulitis and abscess, as well as infection and inflammation of vascular implant. Although some community-acquired bacterial infections in fact are more frequent in the winter time, it is possible that a distinct subset of bacteria, relying less on community transmission, show more virulence in the warmer weather.

Our finding that myasthenia gravis exacerbation has a periodic increase in incidence will be of interest both to clinicians providing care to the patients as well as to immunologists seeking to understand the conditions in which the autoimmune disease is worsened. We suggest that urinary tract infection in particular, as a strong covariate with the condition and as a seasonally linked disease, may have a role to play in exacerbation. It is known that some antibiotics, including those for treatment of urinary tract infection, can worsen the condition. However, as no single correlating factor explains the seasonal pattern clearly, this may be an interesting avenue for further research. There may be an underlying infectious agent causing the immune system flare.

Although ICD-9 codes are primarily recorded for administrative purposes, they have a number of advantages for use in research. Coded data provides a clear categorization of patients and thus is a suitable starting point for well-developed computational methods, as compared to other types of EHR information that require inference of disease state. The ICD-9 represent a wide variety of disease, and, importantly, they are an international standard. Thus, a method developed with our system in New York can easily be applied to the myriad other large EHR datasets across the world. We have shown that mining this type of data can generate highly reproducible findings with clinical significance, implying that code-based EHR studies offer a promising avenue of research.

EHRs are an increasingly rich source of information. In the future, projects such as the EMERGE network promise the integration of this phenotypic data with genotypic information [28]. With the advent of these resources, patients who display increased encounters for a disease could be interrogated for genotypic markers, allowing us to find new mechanisms of disease, as has been previously investigated in psychiatric disorders. The appropriate methods to analyze this complex source of information in an unbiased fashion holds great promise for human disease.

## Methods

### Quantifying incidence of diagnoses

We applied LSP-detrend to a de-identified data set from the New York Presbyterian EHR system, previously used in [17]. After reviewing the total number of cases over the entire period recorded in the EHR, we restricted our search to hospital visits happening after 1996, as the number of records dropped significantly before this year. Examining all codes with more than 500 cases in that time period, we obtained patient identifier and date of diagnosis. For each month, the number of unique patients presenting with the diagnosis was recorded and used as the input to the subsequent analyses.

### Correcting for confounding trends

We added the number of cases of each diagnosis together for each month to examine the aggregate trend of hospital visits. As described in Results and shown in Figure 1A, the number of cases increases steadily over time. As periodograms evaluate how the change from the mean signal depends on a function of time, these larger changes obscure the smaller scale periodic pattern: before trend removal, no seasonal pattern is detected, but after the trend is removed, the pattern is evident (Figure 1B). Thus, we subtract out a smoothed version of the data in order to remove the overall trend. The trends actually are different for every diagnosis, so we calculate the smoothed data per diagnosis using the kernel density estimation implementation from MATLAB, evaluating the kernel-estimated density at every month, which also estimates the appropriate bandwidth [29]. We remove the months two kernel bandwidths from the beginning and end of the entire time period, as they cannot have reliable density estimates. Then, we subtract out the smoothed estimate from the observations in order to create an incidence data set with no overall trend.

Next, we remove the seasonal hospital visit trend that was also described in the Results. To do this, we make use of the summed number of hospital visits, once its trend is removed. We then estimate the number of diagnoses of a disease per month that would occur if that diagnosis was always a fixed proportion of the total diagnoses, by mean-scaling the total diagnoses. That monthly estimate is subtracted from the de-trended data to remove the seasonal hospital trend.

### Evaluating periodicity

The Lomb-Scargle method for evaluating periodicity was first developed in order to assess periodicity for unevenly time-spaced observations [30,31]. For a time series, it computes a periodogram, evaluating the predictive power of the tested frequency. As the null distribution of the periodogram for a given frequency has been shown to be an exponential, we can assign a statistical significance to a given power [31]. We use a MATLAB implementation of the Lomb-Scargle method, as described by Press, et al [32], with the corrected data described in the previous section. Periodic signal with a period of more than 1.5 years was set to have a non-significant p-value, as these longer periods were less interpretable and less well supported by the detrended data, which only spanned ten years. As we tested 2,805 diseases for periodic patterns of incidence, we use the Benjamini-Hochberg procedure (also implemented in MATLAB), and we found that a Lomb-Scargle p-value of < .01 had an expected false discovery rate of less than 15%.

### Comorbidity analysis

We have previously described ADAMS, an algorithm for identifying comorbid disorders that specifically associated with a given query disease. This method identifies diseases that are more likely to co-occur with the query disease than a given control disease, applying bootstrapping to estimate the false discovery rate. We restricted co-occurring diseases to those occurring within 60 days before the acute exacerbation event, in order to capture factors with likely immediate influence on, or reflection of, a patient's state in the lead-up to the event. For control diseases for myasthenia gravis with acute exacerbation, we selected events with no likely direct link to myasthenia gravis, but that occur in patient groups of similar age and that were frequently diagnosed in this data set., First, we used influenza (code 487.1), as it is a very common disease that strikes a wide range of age groups in the winter. Additionally, we used hip joint pain (719.45) as this condition strikes patients with a similar age distribution as myasthenia gravis, and encounters of this condition increase in the summer. The intersection of the findings from using each control was found, and conditions directly associated with acute exacerbation in particular were removed to create Table 1. The full list of associated conditions is available in Additional File 2.

## List of abbreviations

EHR: Electronic Health Records

ICD-9: International Classification of Diseases, Ninth Revision, Clinical Modification

ADAMS: Application for Discovering Disease Associations using Multiple Sources

## Competing interests

The authors declare that they have no competing interests.

## Authors' contributions

R.D.M. developed the methods, performed the analysis, and drafted the manuscript. H.K. developed the methods and revised the manuscript. R.R. conceived of the method, participated in its design, and helped to draft the manuscript. All authors read and approved the final manuscript.

## Supplementary Material

Additional file 2 supp_table_2.xls. This is is the extension of Table 1, showing all associated health conditions, including those that are most likely the result of myasthenia gravis exacerabation. The shaded entries are likely results of the exacerbation. Click here for file

Additional file 1 **supp_table_1**.xls. For 284 codes with significant periodicity once trend is removed, the period and the p-value are shown for each code. Click here for file

## Declarations

This work has been funded by NIH grants U54 CA121852-05 and P50 MH094267-01 and the Conte Center for Neuropsychiatric Genomics grant. Publication for this article has been funded by the Conte Center for Neuropsychiatric Genomics grant.

This article has been published as part of *BMC Bioinformatics *Volume 15 Supplement 6, 2014: Knowledge Discovery and Interactive Data Mining in Bioinformatics. The full contents of the supplement are available online at http://www.biomedcentral.com/bmcbioinformatics/supplements/15/S6.
